# Supplementary material for: Association between Ambient Temperature and Severe Diarrhoea in the National Capital Region, Philippines
Source: Int J Environ Res Public Health. 2021 Aug 2;18(15):8191. doi: 10.3390/ijerph18158191 (PMC8346076; doi:10.3390/ijerph18158191)
Supplement: Supplementary file 1 [file ijerph-18-08191-s001.zip › ijerph-1277832-supplementary.pdf]

Association between ambient temperature and severe diarrhoea in the National Capital Region, Philippines

Supplemental

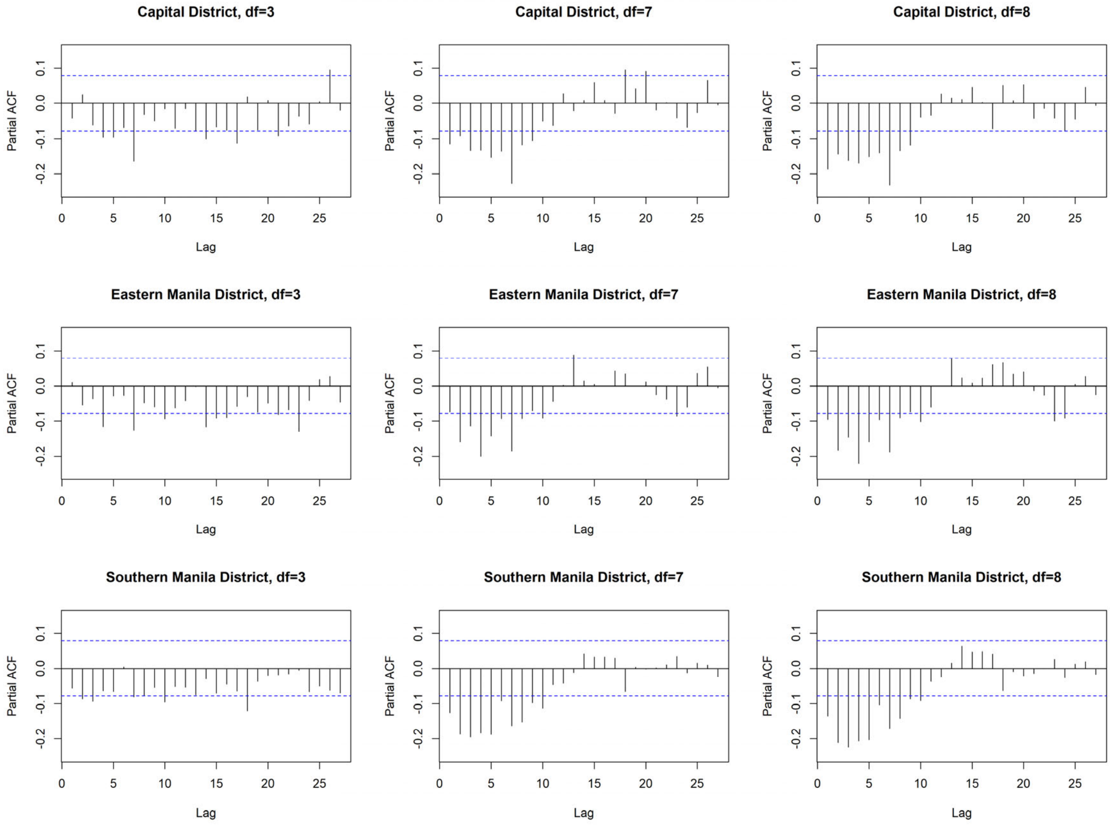

Figure S1. Partial autocorrelation of models for associations between mean temperature and mortality due to diarrhoea in three NCR districts by increasing degrees of freedom for week (multiplied by 12 years).

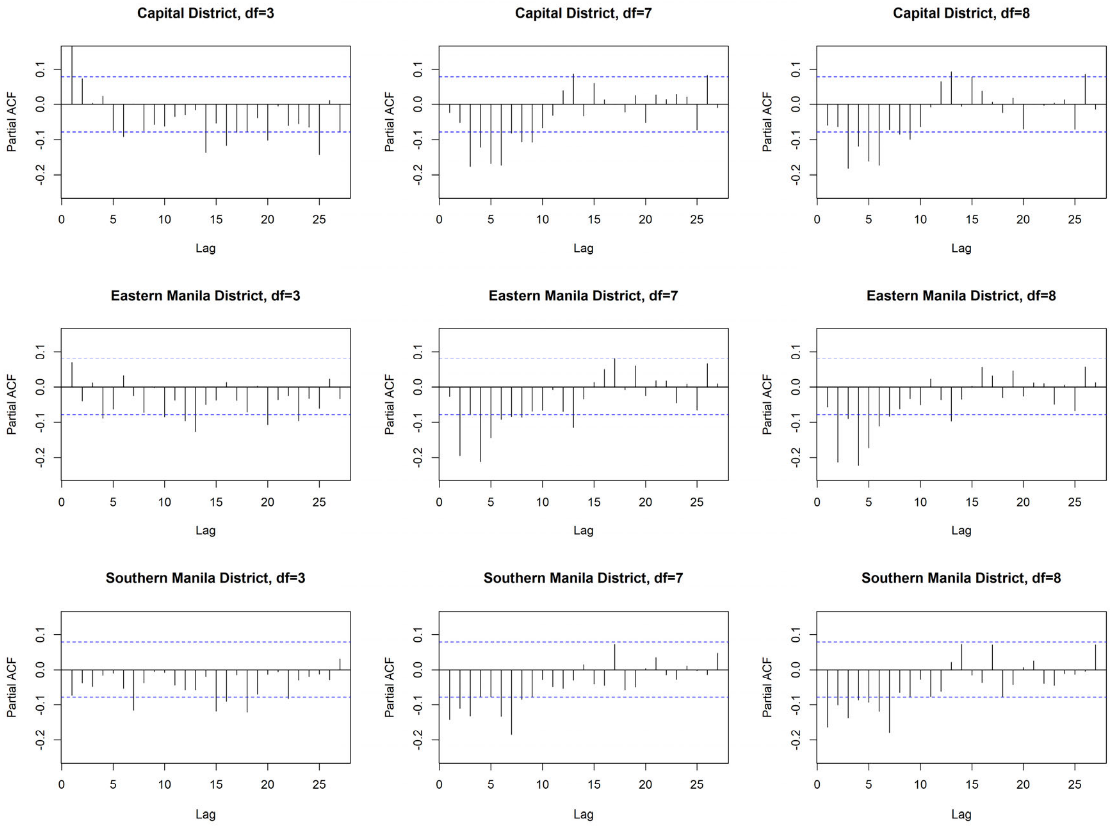

Figure S2. Partial autocorrelation of models for associations between mean temperature and hospitalised severe diarrhoea cases in three NCR districts by increasing degrees of freedom for week (multiplied by 12 years).

Table S1. ICD 10 codes of mortality due to diarrhoea in three NCR districts in 2006-2017.

ICD 10 codes	Capital District (n=1183)	Eastern Manila (n=2020)	Southern Manila (n=1983)	NCR (n=5186)
A00: Cholera	2 (0.17%)	1 (0.05%)	3 (0.15%)	6 (0.12%)
A01: Typhoid and paratyphoid fevers	40 (3.38%)	71 (3.51%)	68 (3.43%)	179 (3.45%)
A02: Other salmonella infections	1 (0.08%)	2 (0.10%)	2 (0.10%)	5 (0.10%)
A03: Shigellosis	1 (0.08%)	0 (0.00%)	1 (0.05%)	2 (0.04%)
A04: Other bacterial intestinal infections	5 (0.42%)	5 (0.25%)	6 (0.30%)	16 (0.31%)
A05: Other bacterial foodborne intoxications	0 (0.00%)	0 (0.00%)	1 (0.05%)	1 (0.02%)
A06: Amoebiasis	28 (2.37%)	118 (5.84%)	76 (3.83%)	222 (4.28%)
A07: Other protozoal intestinal diseases	1 (0.08%)	0 (0.00%)	1 (0.05%)	2 (0.04%)
A08: Viral and other specified intestinal infections	2 (0.17%)	0 (0.00%)	1 (0.05%)	3 (0.06%)
A09: Other gastroenteritis and colitis of infectious and unspecified origin	1103 (93.23%)	1823 (90.25%)	1824 (91.89%)	4750 (91.59%)

Table S2. ICD 10 codes of hospitalised severe diarrhoea cases in three NCR districts in 2006-2017.

ICD 10 codes	Capital District (n=4122)	Eastern Manila (n=923)	Southern Manila (n=623)	NCR (n=5668)
A00: Cholera	0 (0.00%)	0 (0.00%)	0 (0.00%)	0 (0.00%)
A01: Typhoid and paratyphoid fevers	153 (3.71%)	37 (4.01%)	32 (5.14%)	222 (3.92%)
A02: Other salmonella infections	0 (0.00%)	0 (0.00%)	1 (0.16%)	1 (0.02%)
A03: Shigellosis	3 (0.7%)	0 (0.00%)	0 (0.00%)	3 (0.05%)
A04: Other bacterial intestinal infections	6 (0.15%)	4 (0.43%)	0 (0.00%)	10 (0.18%)
A05: Other bacterial foodborne intoxications	0 (0.00%)	0 (0.00%)	0 (0.00%)	0 (0.00%)
A06: Amoebiasis	315 (7.64%)	79 (8.56%)	33 (5.30%)	427 (7.53%)
A07: Other protozoal intestinal diseases	0 (0.00%)	0 (0.00%)	0 (0.00%)	0 (0.00%)
A08: Viral and other specified intestinal infections	27 (0.66%)	3 (0.32%)	1 (0.16%)	31 (0.55%)
A09: Other gastroenteritis and colitis of infectious and unspecified origin	3618 (87.77%)	800 (86.67%)	556 (89.25%)	4974 (87.76%)

Table S3. Attendance to mortality due to diarrhoea in three NCR districts in 2006-2017.

Attendance	Capital District (n=1183)	Eastern Manila (n=2020)	Southern Manila (n=1983)	NCR (n=5186)
Hospital authority	800 (67.62%)	1264 (62.57%)	1124 (56.68%)	3188 (61.47%)
Private physician	55 (4.65%)	172 (8.51%)	98 (4.94%)	325 (6.27%)
Public health officer	9 (0.76%)	20 (0.99%)	38 (1.92%)	67 (1.29%)
None	317 (26.80%)	563 (27.87%)	719 (36.26%)	1599 (30.83%)
Not stated	0 (0.00%)	1 (0.05%)	0 (0.00%)	1 (0.02%)
Others	2 (0.17%)	0 (0.00%)	4 (0.20%)	6 (0.12%)

Table S4. Age groups of mortality due to diarrhoea in three NCR districts in 2006–2017.

Age groups	Capital District (n=1183)	Eastern Manila (n=2020)	Southern Manila (n=1983)	NCR (n=5186)
<1	325 (27.47%)	542 (26.83%)	585 (29.50%)	1452 (28.00%)
1–4	305 (25.78%)	508 (25.15%)	505 (25.47%)	1318 (25.41%)
5–14	78 (6.59%)	142 (7.03%)	156 (7.87%)	376 (7.25%)
15–44	153 (12.93%)	312 (15.45%)	280 (14.12%)	745 (14.37%)
45–64	136 (11.50%)	204 (10.10%)	204 (10.29%)	544 (10.49%)
65>	184 (15.55%)	300 (14.85%)	253 (12.76%)	737 (14.21%)

Table S5. Age groups of hospital admissions due to severe diarrhoea in three NCR districts in 2006–2017.

Age groups	Capital District (n=4122)	Eastern Manila (n=923)	Southern Manila (n=623)	NCR (n=5668)
<1	1083 (26.27%)	204 (22.10%)	202 (32.42%)	1489 (26.27%)
1–4	1529 (37.09%)	346 (37.49%)	233 (37.40%)	2108 (37.19%)
5–14	490 (11.89%)	124 (13.43%)	68 (10.91%)	682 (12.03%)
15–44	629 (15.26%)	187 (20.26%)	74 (11.88%)	890 (15.70%)
45–64	248 (6.02%)	38 (4.12%)	33 (5.30%)	319 (5.63%)
65>	143 (3.47%)	24 (2.60%)	13 (2.09%)	180 (3.18%)

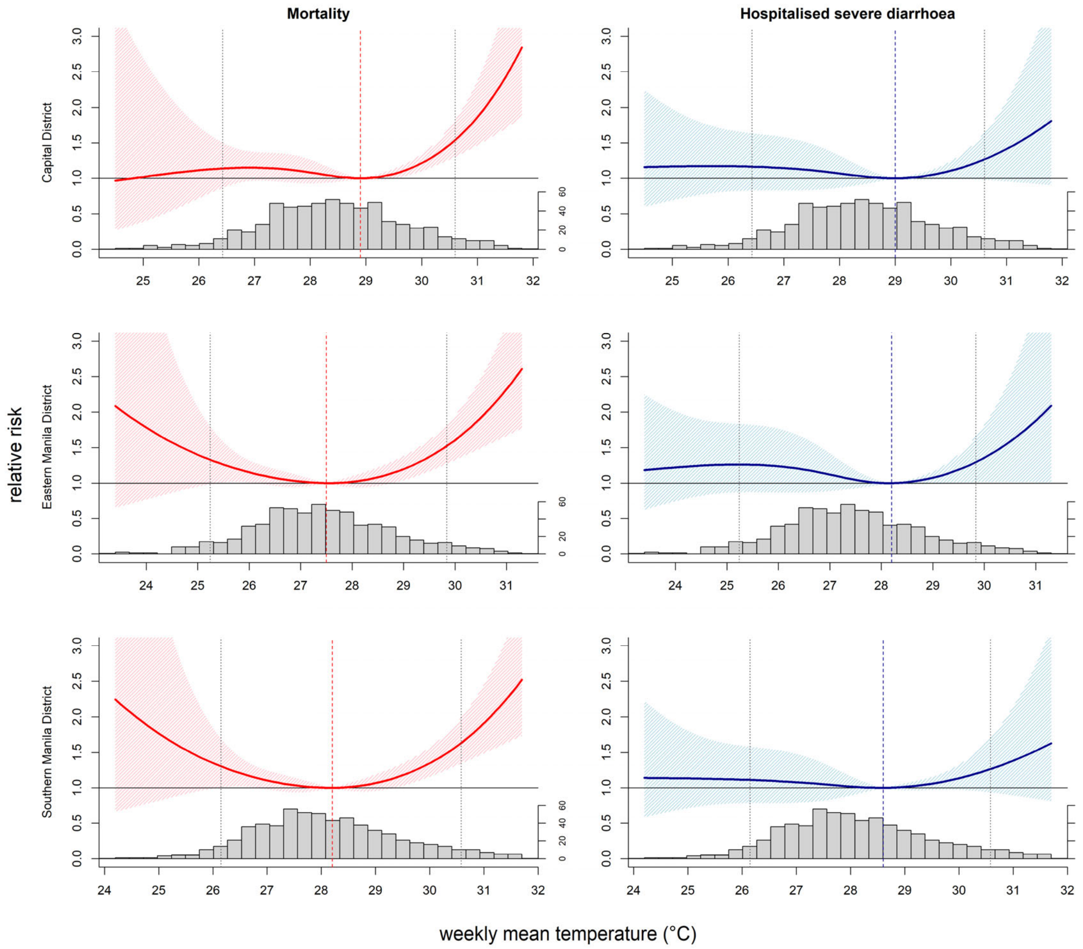

Figure S3. Cumulative associations between mean temperature and severe diarrhoea by district in the National Capital Region of the Philippines in 2006–2017. Curves are the relative risks, polygons are the 95% confidence intervals, vertical dashed lines are the minimum risk temperatures, grey dotted lines are 5th and 95th temperature percentiles, and grey histograms are the temperature distributions.

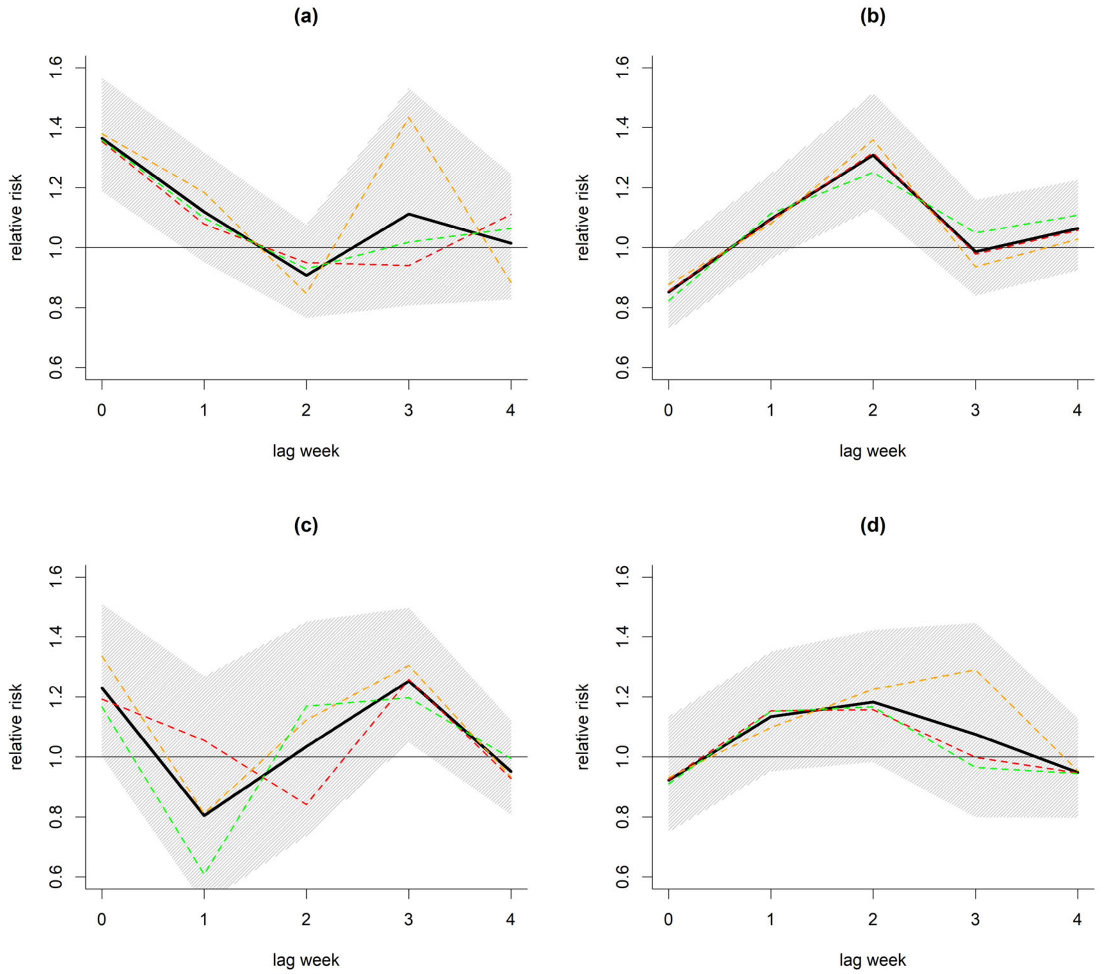

Figure S4. Predictor-specific summary associations between mean temperature and severe diarrhoea in three districts of the National Capital Region in 2006–2017. Mortality risks at high temperature (95th percentile = 30.4°C) (a) and at low temperature (5th percentile = 25.8°C) (b) with minimum risk temperature (MRT) of 28.2°C. Hospitalised severe diarrhoea risks at high temperature (c) and at low temperature (d) with MRT of 28.6°C. Black lines are NCR level relative risks (RR) with grey polygons as 95% confidence intervals. The broken lines refer to RRs of Capital (red), Eastern Manila (orange), and Southern Manila (green) districts.

Table S6. Heterogeneity from meta-regression models for associations between mean temperature and severe diarrhoea in three districts of the National Capital Region in 2006–2017.

Diarrhoea outcome	Minimum risk temperature (°C)	High or low temperature (°C)	I^2 (%)	Cochran Q (*p*-value)
Mortality	28.2	30.4	14.9	11.75 (0.30)
		25.8	0.0	8.08 (0.62)
Hospitalised severe cases	28.6	30.4	15.1	11.77 (0.30)
		25.8	0.0	6.92 (0.73)

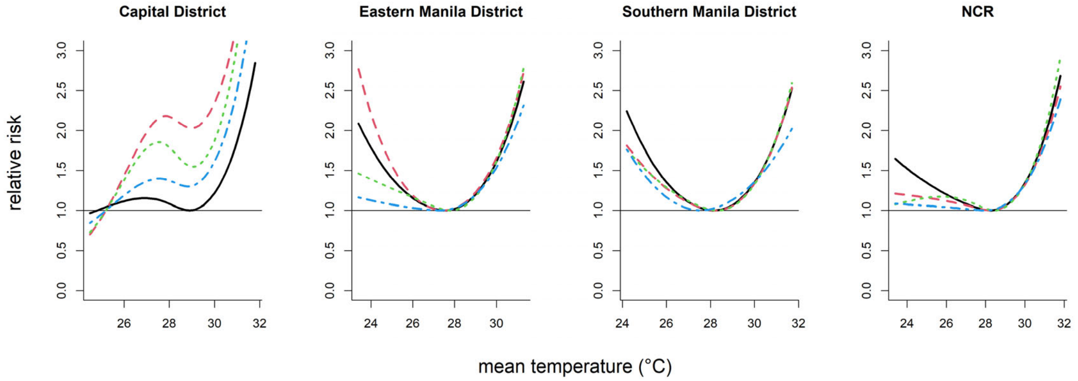

Figure S5. Sensitivity analysis of cumulative associations between temperature and mortality in three NCR districts in 2006-2017 by increasing maximum lags. Black lines refer to 4-week lag, red lines are 5-week lag, green lines are 6-week lag, and blue lines are 7-week lag.

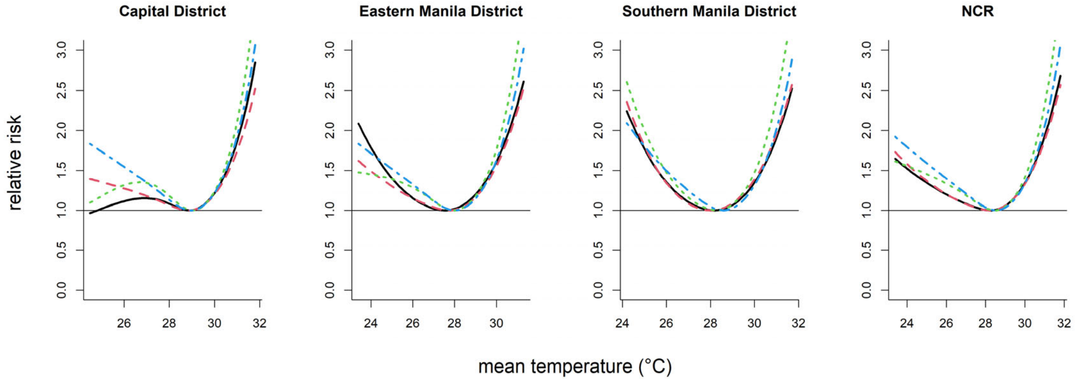

Figure S6. Sensitivity analysis of cumulative associations between temperature and mortality in three NCR districts in 2006-2017 by changing degrees of freedom (df) for week. Black lines refer to 3 df, red lines are 4 df, green lines are 5 df, and blue lines are 6 df.

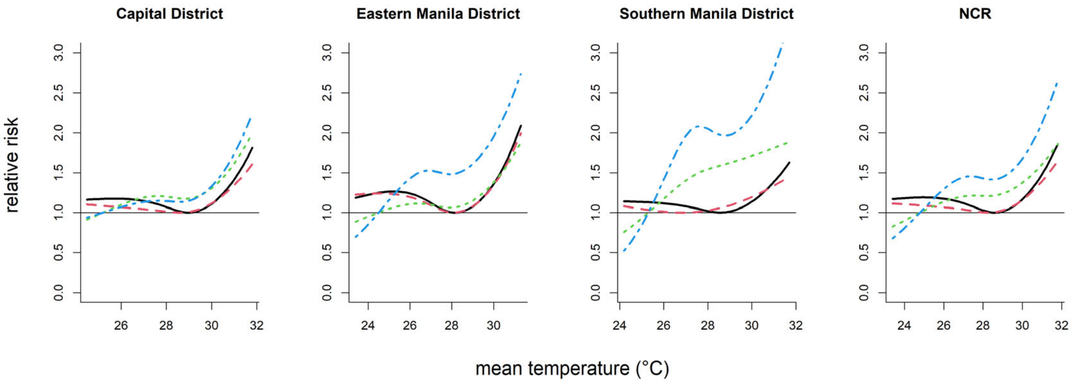

Figure S7. Sensitivity analysis of cumulative associations between temperature and hospitalised severe cases in three NCR districts in 2006-2017 by increasing maximum lags. Black lines refer to 4-week lag, red lines are 5-week lag, green lines are 6-week lag, and blue lines are 7-week lag.

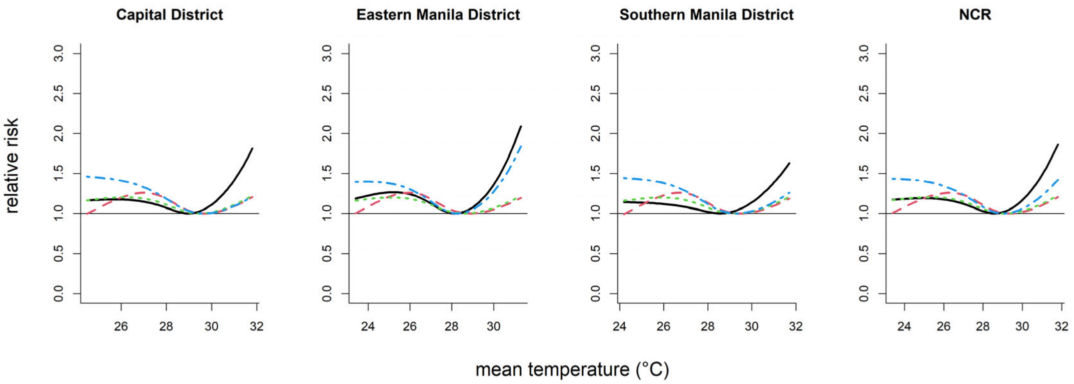

Figure S8. Sensitivity analysis of cumulative associations between temperature and hospitalised severe cases in three NCR districts in 2006-2017 by changing degrees of freedom (df) for week. Black lines refer to 3 df, red lines are 4 df, green lines are 5 df, and blue lines are 6 df.

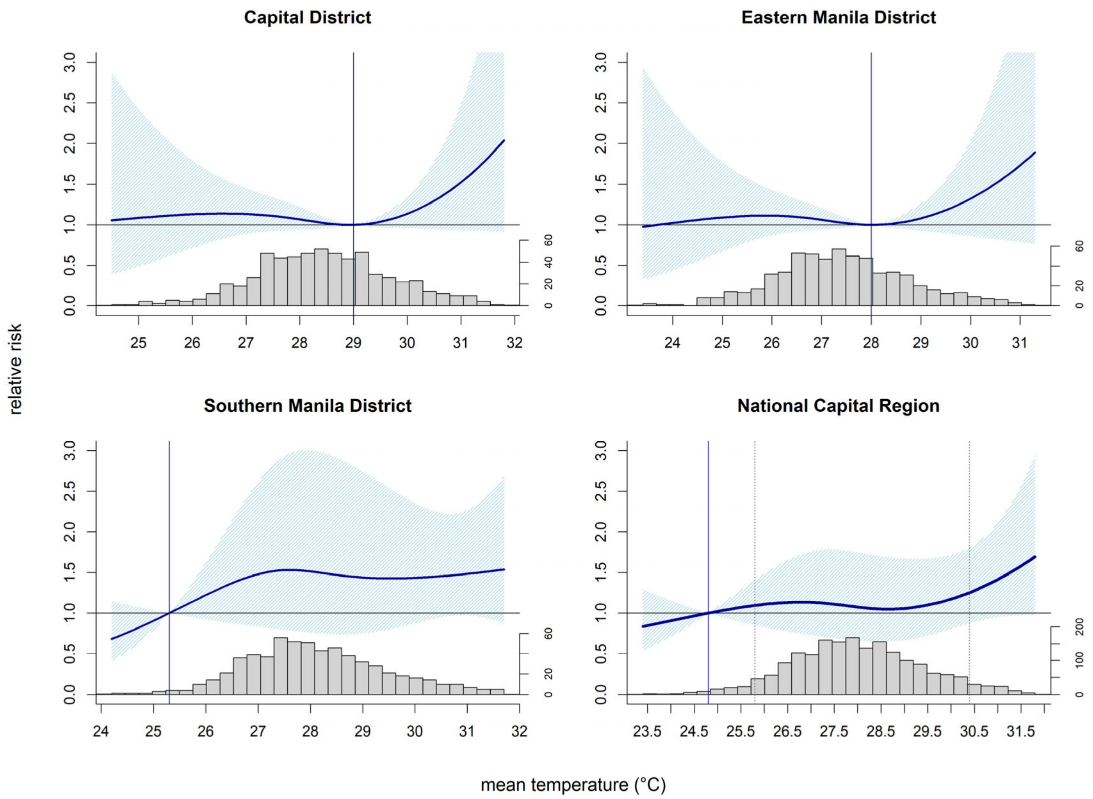

Figure S9. Cumulative associations for hospital admissions due to diarrhoea with mean temperatures in three National Capital Region districts in 2006-2017. Blue curves show the relative risks, blue polygons show the 95% confidence intervals, blue lines are the minimum risk temperatures, dotted lines are 5th and 95th percentile temperatures, and histograms are the temperature distributions.

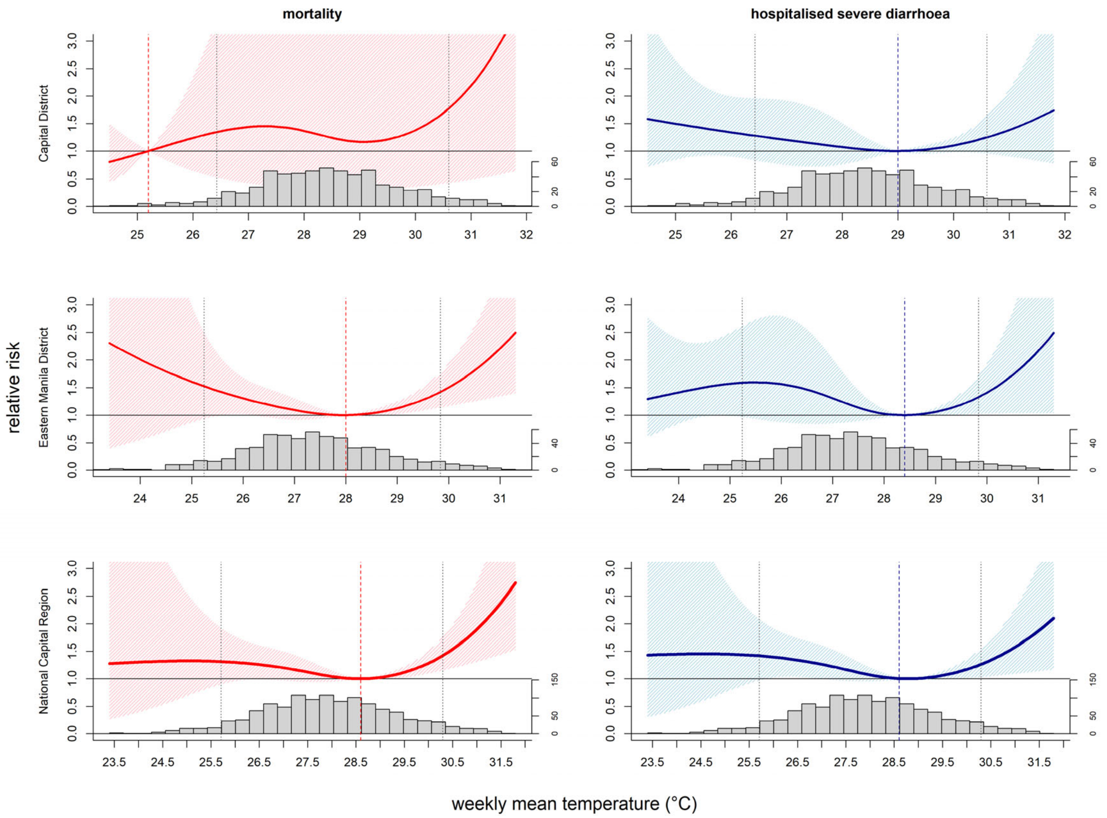

Figure S10. Cumulative associations for mean temperature and severe diarrhoea in the National Capital Region in 2006–2017 (controlling for rainfall). Curves are the relative risks, polygons are the 95% confidence intervals, vertical dashed lines are the minimum risk temperatures, grey dotted lines are 5th and 95th temperature percentiles, and grey histograms are the temperature distributions.
